# Liposomal Glutathione Exhibits Direct and Intracellular Antimycobacterial Activity Against *Mycobacterium avium* via Membrane Depolarization in a THP-1 Macrophage Model

**DOI:** 10.3390/bioengineering13070823

**Published:** 2026-07-17

**Authors:** Nezam Newman, Kayvan Sasaninia, Wajiha Akif, Kaylee Dillard, Destiny Jaime, Kaitlyn Nguyen, Malina Navarrette, Jesse Melendez, Iffat Hasnin Era, Ama Xu, Navya Sharma, Syed Muzzammil Ahmad, Rakesh Kumar Tiwari, Vishwanath Venketaraman

**Affiliations:** 1 Department of Biomedical Sciences, College of Osteopathic Medicine of the Pacific, Western University of Health Sciences, Pomona, CA 91766, USA; nezam.newman@westernu.edu (N.N.); syedahmad@westernu.edu (S.M.A.); 2 College of Health Sciences, Western University of Health Sciences, Pomona, CA 91766, USAkaitlyn.nguyen@westernu.edu (K.N.); 3 College of Natural and Agricultural Sciences, University of California, Riverside, CA 92521, USA; 4 Crean College of Health Sciences, Chapman University, Orange, CA 92866, USA; 5 Department of Biomedical Sciences, Heatherington College of Osteopathic Medicine, Western University of Health Sciences, Lebanon, OR 97355, USA; rtiwari@westernu.edu

**Keywords:** liposomal glutathione, *Mycobacterium avium*, antimycobacterial, membrane depolarization, THP-1 macrophages, nontuberculous mycobacteria

## Abstract

*Mycobacterium avium* (*M. avium*) is an opportunistic intracellular pathogen causing chronic pulmonary disease in immunocompromised individuals, particularly in those with HIV/AIDS. Current treatments present challenges due to duration, antibiotic resistance and toxicity, creating a need for novel treatments. Glutathione (GSH), a key intracellular antioxidant, is depleted in immunocompromised individuals. This study evaluated the direct and intracellular antimycobacterial activity of liposomal glutathione (L-GSH) against *M. avium*. Furthermore, the minimum inhibitory concentration (MIC) and depolarization effects of L-GSH were also determined. Our results indicate that L-GSH has potent direct antimycobacterial activity against *M. avium* through membrane depolarization and reduces intracellular survival of *M. avium* in human macrophages. Given the established GSH deficiency in HIV-infected individuals and the clinical need for novel *Mycobacterium avium* complex (MAC) therapies with favorable safety profiles, these findings support further investigation of L-GSH as a host-directed therapeutic agent for HIV-associated *M. avium* infections.

## 1. Introduction

*Mycobacterium avium* complex (MAC) is a group of nontuberculous mycobacteria (NTM) ubiquitously present in water, soil, and other environmental reservoirs [1,2,3,4,5]. Transmission to humans occurs primarily through inhalation of aerosolized bacteria, leading to pulmonary colonization and infection, particularly in immunocompromised individuals [6,7]. Populations at increased risk include those with HIV/AIDS, chronic obstructive pulmonary disease, cystic fibrosis, and diabetes mellitus [2,3,4,5,8].

### 1.1. MAC as an Opportunistic Pathogen in HIV Infection

Among HIV-infected individuals, MAC is a leading cause of nontuberculous mycobacterial disease and continues to contribute to significant morbidity and mortality despite the widespread use of antiretroviral therapy (ART) [9,10,11,12]. Before the ART era, disseminated MAC infection occurred in up to 40% of AIDS patients with CD4 counts below 50 cells/µL [13]. While ART has dramatically reduced the incidence of disseminated MAC, pulmonary MAC disease remains prevalent, and subclinical immune dysfunction persists in many individuals with HIV even after viral suppression [2,3,4,5,14].

**Figure 1:**
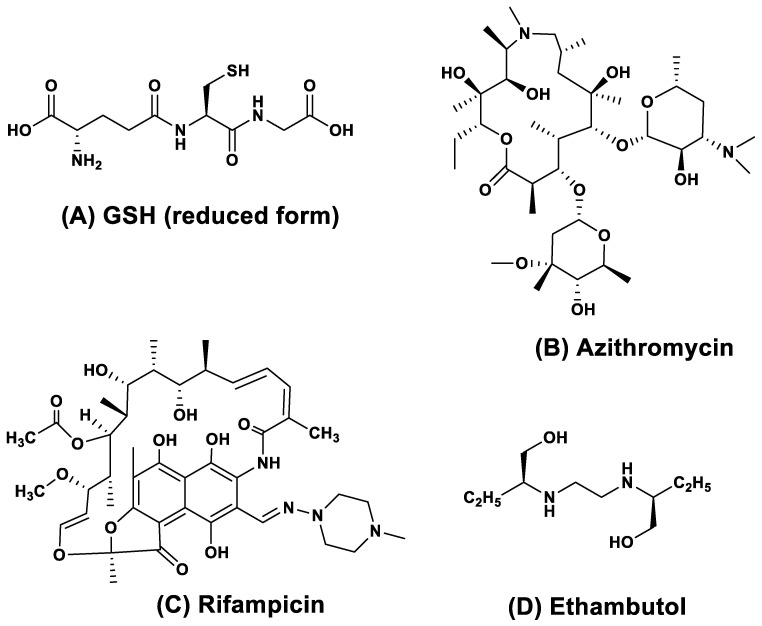
Chemical structures of GSH and standard antimycobacterial agents used in *M. avium* treatment. (**A**) Reduced glutathione (GSH), showing the gamma-glutamyl-cysteinyl-glycine tripeptide backbone and free thiol group responsible for antioxidant and membrane-interacting activity. (**B**) Azithromycin, a macrolide that inhibits the 50S ribosomal subunit. (**C**) Rifampicin, an ansamycin that inhibits RNA polymerase. (**D**) Ethambutol, a cell wall synthesis inhibitor targeting arabinosyl transferases. The structural and mechanistic distinction between GSH and standard antimycobacterial agents supports its potential as a host-directed adjunct.

Once inhaled, *M. avium* is phagocytosed by alveolar macrophages, where it resides in modified phagosomes and resists killing mechanisms, including reactive oxygen and nitrogen intermediates [15,16]. The bacterium produces biofilms and possesses a lipid-rich cell wall containing glycopeptidolipids that contribute to intrinsic antibiotic resistance [17,18]. Current treatment guidelines recommend a 12-month or longer multi-drug regimen typically including a macrolide, rifampicin, and ethambutol [7,19] (Figure 1). However, treatment success rates remain low due to poor adherence, drug toxicity (e.g., ethambutol-induced optic neuritis), and emerging resistance [20,21]. Additionally, rifampicin-containing regimens pose significant drug–drug interactions with many antiretroviral agents, complicating co-management of HIV and MAC [22,23,24]. There is therefore an urgent need for novel therapeutic strategies that are effective, well-tolerated, and compatible with ART.

### 1.2. Glutathione Deficiency in HIV Infection: Mechanisms and Immune Consequences

Glutathione (GSH; γ-glutamyl-cysteinyl-glycine, Figure 1A) is the principal intracellular antioxidant and plays a critical role in maintaining redox balance and immune cell function [25,26]. GSH deficiency has been well documented in HIV-infected individuals. Herzenberg and colleagues demonstrated that HIV-infected individuals have significantly lower GSH levels in CD4+ T cells, CD8+ T cells, and monocytes compared to healthy controls, and that this deficiency correlates with impaired survival [27,28,29].

Subsequent studies from our laboratory as well as other laboratories have elucidated the mechanisms underlying GSH deficiency in HIV infection [30,31,32,33,34,35,36,37,38,39,40,41,42,43,44,45,46,47,48,49,50,51]. Morris et al. (2012) observed that macrophages from HIV-positive individuals have lower intracellular GSH levels, with the GSH composition heavily favoring oxidized glutathione (GSSG) over free reduced GSH (rGSH). This deficiency correlated with increased growth of *Mycobacterium tuberculosis* inside macrophages [30,31,32]. Furthermore, Morris et al. (2014) demonstrated that the levels of enzymes responsible for GSH synthesis, glutathione synthase (GSS), glutamate-cysteine ligase catalytic subunit (GCLC), and glutathione reductase (GSR) are significantly reduced in erythrocytes from HIV-infected individuals, directly linking compromised GSH levels to decreased expression of GSH-synthetic enzymes [33,34,35,36]. Saing et al. (2016) confirmed these findings in brain tissue, showing that GCLC, GSS, and GSR are significantly decreased in HIV-infected individuals, particularly those with low CD4 counts (<200 cells/mm^3^) [37].

The functional consequences of GSH deficiency are profound [38,39,40]. Guerra et al. (2011) reported that T lymphocytes from HIV-infected individuals are deficient in GSH, and this deficiency correlates with decreased levels of Th1 cytokines (IL-2, IL-12, IFN-γ) and enhanced growth of mycobacteria inside human macrophages [38]. Morris et al. (2012) and Valdivia et al. (2017) further demonstrated increased levels of free radicals and altered cytokine profiles in HIV-infected individuals, characterized by increased pro-inflammatory and immunosuppressive mediators [30,31,32,41,42,43]. This Th2-biased immune profile compromises protection against intracellular pathogens like *M. avium*.

Importantly, restoration of intracellular GSH levels through supplementation has been shown to reverse these defects. Ly et al. (2015) demonstrated that supplementing HIV-infected individuals with liposomal GSH (L-GSH) for 13 weeks resulted in a significant increase in Th1 cytokines (IL-1β, IL-12, IFN-γ, TNF-α) and a substantial decrease in free radicals and immunosuppressive cytokines (IL-10, TGF-β) [44,45]. Valdivia et al. (2017) confirmed these findings in a double-blinded study, showing that L-GSH supplementation for 3 months in HIV-infected individuals with low CD4 counts restored redox homeostasis and cytokine balance [41]. Morris et al. (2013) further showed that treatment with N-acetylcysteine or L-GSH replenished rGSH levels in macrophages from HIV-infected individuals and correlated with decreased intracellular growth of *M. tuberculosis* [46,47,48].

### 1.3. Liposomal Glutathione as a Therapeutic Strategy for HIV-Associated MAC

Free GSH is poorly absorbed and rapidly degraded in the gastrointestinal tract. GSH encapsulated in liposomes (L-GSH) has been shown to improve cellular delivery and bioavailability [52]. Our laboratory has demonstrated that L-GSH supplementation enhances macrophage control of mycobacterial infections and improves immune responses in HIV-infected individuals [41,42,43,44,45,46,47,48,53].

Nikjeh et al. (2025) specifically highlights host-directed strategies, including GSH augmentation, as promising adjuncts for managing HIV-associated mycobacterial diseases, including MAC, with favorable ART compatibility [10,11,12]. Unlike rifampicin-containing regimens that interact with protease inhibitors and non-nucleoside reverse transcriptase inhibitors [22,23,24], L-GSH has no known significant drug–drug interactions with antiretroviral agents [44,45,49,50,51].

Given the established impairment of GSH homeostasis in HIV-infected individuals and the high susceptibility of this population to disseminated MAC disease, these findings have important translational implications and support further evaluation of GSH-based interventions as adjunctive therapies to enhance host immunity and improve infection control.

Prior studies from this laboratory established GSH-dependent immune control of *M. tuberculosis* and restored Th1 cytokine profiles in HIV-infected individuals through oral L-GSH supplementation. Whether L-GSH has direct, immune cell-independent bactericidal activity against *M. avium*, the predominant NTM pathogen in HIV infection, has not been examined. This study demonstrates, for the first time, that L-GSH exerts concentration- and time-dependent direct antimycobacterial activity against *M. avium* through membrane depolarization and reduces intracellular bacterial survival in human macrophages at concentrations relevant to the intracellular compartment.

## 2. Materials and Methods

### 2.1. Bacterial Strain and Culture Conditions

*M. avium*, utilized in the study, was derived from ATCC 25291^TM^, obtained via KWIK-STIK^TM^ (Microbiologics, St. Cloud, MN, USA). It was cultured in Middlebrook 7H9 broth (BD Biosciences, Franklin Lakes, NJ, USA) supplemented with 10% albumin-dextrose complex (ADC; 50 g/L bovine serum albumin, 20 g/L dextrose, 8.5 g/L NaCl), 0.5% glycerol, and 0.05% Tween 80. Cultures were incubated at 37 °C until reaching logarithmic growth phase, defined as an optical density at 600 nm (OD_600_) of 0.5–0.8. For solid medium, Middlebrook 7H11 agar (Sigma-Aldrich, St. Louis, MO, USA) supplemented with 10% ADC and 0.5% glycerol was used.

### 2.2. Processing of M. avium for Experiments

*M. avium* cultures were centrifuged at 3000× *g* for 15 min at room temperature. The bacterial pellet was washed twice and resuspended in sterile phosphate-buffered saline (PBS; pH 7.4). Bacterial clumps were disaggregated by vortexing at 3 min intervals with 3 mm sterile glass beads. *M. avium* suspension was then passed through a 5 µm syringe filter to obtain a single-cell mycobacterial suspension. The processed bacterial cell suspension was serially diluted and plated on 7H11 agar to determine *M. avium* numbers in colony-forming unit (CFU).

### 2.3. Liposomal Glutathione (L-GSH)

L-GSH (ReadiSorb) was provided by Your Energy Systems, Palo Alto, CA, USA. Working concentrations were prepared by dilution in sterile PBS or culture medium immediately before use. The concentrations tested for direct antimycobacterial activity were 10 mM, 20 mM, and 40 mM. For THP-1 intracellular studies, concentrations of 1 mM, 2 mM, 4 mM (low range) and 10 mM, 20 mM, 40 mM (high range) were used. The reduced form of free glutathione (rGSH; Sigma-Aldrich, St. Louis, MO, USA) was used as a comparator in membrane depolarization experiments at 40 mM.

### 2.4. Direct Antimycobacterial Activity Assay (CFU)

*M. avium* was cultured in 7H9 medium and treated with L-GSH (10–40 mM) for up to 8 days. Viability was quantified by CFU. Processed *M. avium* was diluted to approximately 1 × 10^5^ CFU/mL in 7H9 medium. Aliquots (200 µL) were dispensed into 96-well plates in triplicate. L-GSH was added to achieve final concentrations of 10 mM, 20 mM, and 40 mM. Untreated controls received an equal volume of PBS. Plates were incubated at 37 °C. Treatment groups were terminated at 3 h, 4 days, and 8 days. For the 4-day and 8-day time points, fresh L-GSH was replenished on day 3 and day 6, respectively.

At each termination point, serial 10-fold dilutions of each sample were prepared in PBS. From each dilution, 25 µL was plated onto 7H11 agar plates (divided into halves to plate each sample in duplicate). Plates were incubated at 37 °C for 2–3 weeks until colony growth was observed visually. Colonies were counted, and CFU/mL was calculated.

### 2.5. Minimum Inhibitory Concentration (MIC)

The MIC of L-GSH against *M. avium* was determined using a broth dilution method. *M. avium* at 1 × 10^5^ CFU/mL was treated with L-GSH at concentrations of 600 µM, 800 µM, 1 mM, 2 mM, and 4 mM in 96-well plates in triplicate. Untreated controls received PBS. Plates were incubated at 37 °C for 4 days. Fresh L-GSH was replenished on day 3. Bacterial growth was assessed by measuring OD_600_ before and after incubation. Percent inhibition was calculated using the formula [54]:

(1) $$\%\;Inhibition=[\frac{{(OD}_{600}\;Control-\;{(OD}_{600}\;T2-{OD}_{600}\;T1))}{{OD}_{600}\;Control}]\times 100\%$$

where

- OD_600_ Control = Optical density of untreated bacteria at the end of the incubation period measured directly from the control well at termination.
- OD_600_ T1 = Optical density of treated bacteria at the start of incubation (Day 0, before treatment) measured from the treated well at time zero.
- OD_600_ T2 = Optical density of treated bacteria at the end of incubation measured from the treated well at termination.

Because *M. avium* grows as clumps in broth culture, visual assessment of growth inhibition is unreliable. We therefore determined MIC defined as the lowest concentration achieving at least 50% OD-based growth inhibition with consistent reproducibility across three independent experiments (SD < 10% of the mean). This operational definition reflects the growth-inhibitory threshold under the conditions of this assay and is reported as such throughout.

### 2.6. Membrane Depolarization Assay (DiSC_3_(5))

Membrane depolarization was assessed using the fluorescent probe DiSC_3_(5) (3,3′-dipropylthiadicarbocyanine iodide; Sigma-Aldrich). *M. avium* was harvested, washed, and resuspended in buffer (5 mM HEPES, 20 mM glucose, 100 mM KCl, pH 7.2) to an OD_600_ of 0.1. DiSC_3_(5) was added to a final concentration of 0.5 µM, and bacteria were incubated until stable fluorescence was achieved (approximately 30 min). Carbonyl cyanide m-chlorophenyl hydrazone (CCCP; 10 µM) served as a positive control for membrane depolarization. L-GSH (40 mM) and rGSH (40 mM) were added, and fluorescence (excitation 622 nm, emission 670 nm) was monitored over 60 min using a fluorescence plate reader (SpectraMax iD3, Molecular Devices, San Jose, CA, USA). Increased fluorescence indicates membrane depolarization.

### 2.7. THP-1 Cell Culture and Differentiation

Human THP-1 monocytic cells (ATCC TIB-202, Manassas, VA, USA) were maintained in RPMI 1640 medium containing 2 mM L-glutamine (Sigma-Aldrich) and supplemented with 10% heat-inactivated fetal bovine serum (FBS) at 37 °C in 5% CO_2_. Cells were passaged every 3–4 days and maintained at a density between 2 × 10^5^ and 1 × 10^6^ cells/mL by enumerating them using a hemocytometer and trypan blue stain. For differentiation into macrophage-like cells, THP-1 cells were seeded in 96-well plates pre-coated with 0.001% poly-L-lysine at a density of 1 × 10^5^ cells/well. Differentiation was induced by adding phorbol 12-myristate 13-acetate (PMA) at a final concentration of 10 ng/mL for 24 h. Differentiation was confirmed by visualization of cells under an inverted microscope to have macrophage-like nucleus and granular morphology. Following differentiation, media containing PMA was removed and fresh media was added to the wells prior to infection.

### 2.8. THP-1 Infection and Intracellular Survival Assay

We determined the intracellular survival of *M. avium* in THP-1 macrophages treated with L-GSH (1–4 mM and 10–40 mM). Differentiated THP-1 macrophages were infected with processed *M. avium* at a multiplicity of infection (MOI) of 1:1 (bacteria:macrophage) for 1 h at 37 °C in 5% CO_2_. After infection, extracellular bacteria were removed by washing three times with warm PBS. To confirm complete removal of extracellular bacteria, the supernatant from the final wash was collected, serially diluted, and plated onto 7H11 agar in parallel with the intracellular lysate samples. No colonies were recovered from final wash supernatants in any experiment, confirming that CFU counts reflect intracellular bacteria only. Infected macrophages were then treated with L-GSH at low concentrations (1 mM, 2 mM, 4 mM) in one experiment and high concentrations (10 mM, 20 mM, 40 mM) in a separate experiment. Untreated infected controls received an equal volume of PBS. All conditions were performed in triplicate. The concentrations used in intracellular experiments (1 to 40 mM) exceed the MIC determined in axenic culture (800 µM) because liposomal drug delivery to intracellular bacteria is subject to barriers not present in broth. After phagocytosis or endocytosis of liposomes, GSH release depends on liposomal fusion with the phagosomal membrane and pH-dependent content release. The effective concentration of GSH reaching *M. avium* within the phagosomal compartment is substantially lower than the nominal extracellular concentration added to the culture well. Higher extracellular concentrations were therefore tested to establish a dose–response relationship across a pharmacologically meaningful intracellular range. Findings from our previous studies indicate that treatment with 40 mM L-GSH resulted in improved control of *M. tuberculosis* infection [55,56].

Treatments were maintained for 3 h, 4 days, or 8 days. For the 4-day and 8-day time points, fresh L-GSH was replenished on day 3 and day 6. At each termination point, supernatants were removed, and cells were washed once with PBS. Macrophages were lysed with 300 µL ice-cold nanopure water, and wells were scraped gently to dislodge cells and release intracellular bacteria. Lysates were serially diluted in PBS and plated onto 7H11 agar as described above. Colonies were counted after visible colony growth was observed following 2–3 weeks of incubation at 37 °C.

### 2.9. Statistical Analysis

All experiments were performed in triplicate and repeated independently at least three times (*n =* 3 independent experiments). Data are presented as mean ± standard error of the mean (SEM). Comparisons between multiple groups were analyzed using one-way analysis of variance (ANOVA). Pairwise comparisons between two groups were analyzed using an unpaired two-tailed Student’s *t*-test. For time-course membrane depolarization data, two-way ANOVA was used. A *p* value < 0.05 was considered statistically significant. Significance levels are denoted as *p* ≤ 0.05, *p* ≤ 0.01, *p* ≤ 0.001, and *p* ≤ 0.0001. Statistical analyses were performed using GraphPad Prism version 9/10 (GraphPad Software, San Diego, CA, USA).

## 3. Results

### 3.1. L-GSH Exerts Direct Antimycobacterial Activity Against M. avium in Axenic Culture

**Figure 2:**
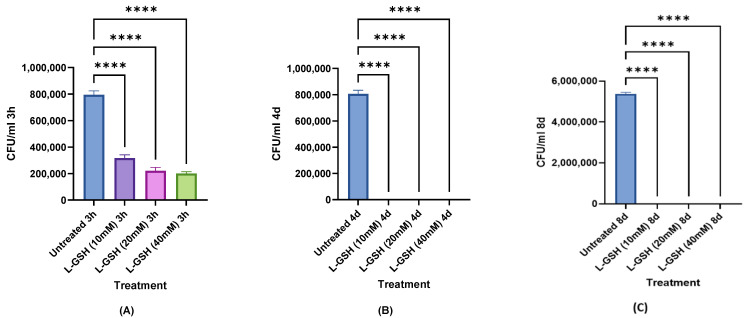
Direct antimycobacterial effects of L-GSH against *M. avium* grown in 7H9 medium. *M. avium* was treated with L-GSH at 10 mM, 20 mM, or 40 mM, and bacterial viability was assessed by CFU enumeration. (**A**) 3 h post-treatment; (**B**) 4 days post-treatment; (**C**) 8 days post-treatment. Data are presented as mean ± SEM from three independent experiments each performed in triplicate (n = 3). Statistical analysis: one-way ANOVA. **** *p* ≤ 0.0001 compared to untreated control.

To determine whether L-GSH has intrinsic antimycobacterial activity independent of host cells, *M. avium* grown in 7H9 medium was treated with L-GSH at concentrations of 10 mM, 20 mM, and 40 mM. Bacterial survival was assessed by CFU enumeration at 3 h, 4 days, and 8 days post-treatment (Figure 2).

At 3 h post-treatment (Figure 2A), L-GSH produced a statistically significant reduction in *M. avium* CFU compared to untreated controls at all concentrations tested (*p* ≤ 0.0001 for 20 mM and 40 mM). The effect was concentration-dependent, with the 40 mM treatment showing the greatest reduction.

At 4 days post-treatment (Figure 2B), a more pronounced antimycobacterial effect was observed. L-GSH significantly suppressed *M. avium* growth across all concentrations compared to untreated controls (*p* ≤ 0.0001 for all). The 40 mM treatment reduced CFU to near the limit of detection.

At 8 days post-treatment (Figure 2C), L-GSH produced sustained and potent antimycobacterial activity. At 40 mM, bacterial CFU were reduced to below the limit of detection, indicating near-complete bacterial clearance. Concentration-dependent reductions were maintained, with 10 mM and 20 mM also showing significant suppression compared to untreated controls (*p* ≤ 0.0001).

These findings demonstrate that L-GSH exerts direct, concentration-dependent, and time-dependent antimycobacterial activity against *M. avium* in axenic culture. The modest effect at 3 h is consistent with the slow generation time of *M. avium* (approximately 24 h) and the kinetics of L-GSH interaction with the mycobacterial outer membrane. Concentration-dependent killing became clearly evident at 4 days and was maintained at 8 days, supporting a time-dependent accumulation of membrane damage rather than rapid bactericidal kinetics typical of fast-acting membrane-disruptive agents.

### 3.2. L-GSH Induces Membrane Depolarization in M. avium

To investigate the mechanism underlying the direct antimycobacterial activity of L-GSH, membrane depolarization was assessed using DiSC_3_(5). Depolarization of the bacterial membrane results in dye release and increased fluorescence. CCCP (10 µM), a protonophore, served as a positive control for complete membrane depolarization.

**Figure 3:**
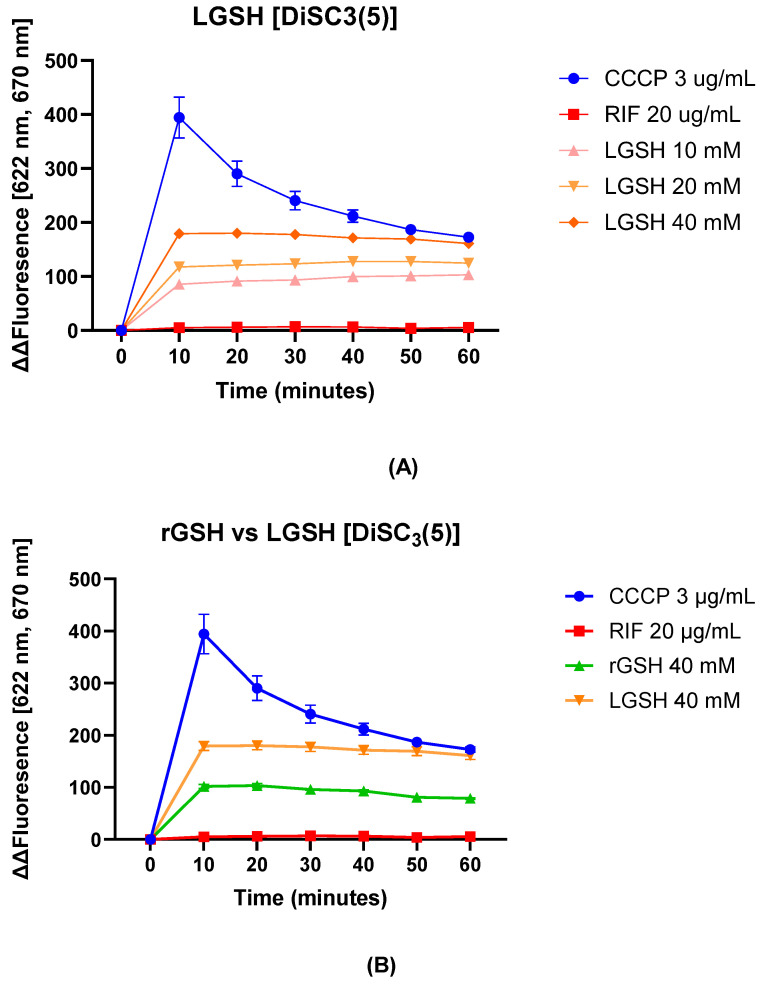
L-GSH induces membrane depolarization in *M. avium*. Membrane potential was assessed using DiSC3(5). CCCP (10 µM) served as a positive control. (**A**) L-GSH (40 mM) induced a time-dependent increase in fluorescence over 60 min compared to untreated and rifampicin-treated controls. (**B**) At 40 mM, L-GSH produced significantly greater membrane depolarization than rGSH. Data are presented as mean ± SEM from three independent experiments performed in triplicate. Statistical analysis: two-way ANOVA. *p* ≤ 0.05, *p* ≤ 0.001, *p* ≤ 0.0001 comparing L-GSH to rGSH at matched time points.

L-GSH (40 mM) induced a time-dependent increase in fluorescence over the 60 min observation period, indicating progressive membrane depolarization (Figure 3A). The depolarization effect was significantly greater than that observed in untreated bacteria and rifampicin-treated controls (which showed minimal membrane disruption).

When compared to rGSH at the same concentration (40 mM), L-GSH produced significantly greater membrane depolarization throughout the 60 min time course (Figure 3B). This suggests that liposomal encapsulation enhances the membrane-disrupting activity of GSH.

These results indicate that membrane depolarization is a contributing mechanism to the direct antimycobacterial activity of L-GSH against *M. avium*.

### 3.3. Minimum Inhibitory Concentration (MIC) of L-GSH Against M. avium

To determine the lowest concentration of L-GSH required to inhibit *M. avium* growth, MIC assays were performed over a concentration range of 600 µM to 4 mM (Table 1). At 600 µM, L-GSH produced modest inhibition (35.27% ± 4.92%). The lowest concentration achieving stable, reproducible inhibition with a standard deviation below 10% was 800 µM (67.81% ± 2.45%). Higher concentrations (1–4 mM) produced progressively greater inhibition, with 4 mM achieving 108.76% ± 0.67% inhibition, indicating bactericidal activity. The MIC was therefore defined as 800 µM (0.8 mM), the lowest concentration producing sustained growth inhibition relative to untreated controls under the conditions of this assay.

### 3.4. L-GSH Reduces Intracellular M. avium Survival in THP-1 Macrophages

To determine whether L-GSH retains antimycobacterial activity in a host cell environment, differentiated THP-1 macrophages were infected with *M. avium* and treated with L-GSH. Intracellular bacterial survival was assessed at 3 h, 4 days, and 8 days post-infection.

#### 3.4.1. Low Concentration Range (1–4 mM)

**Figure 4:**
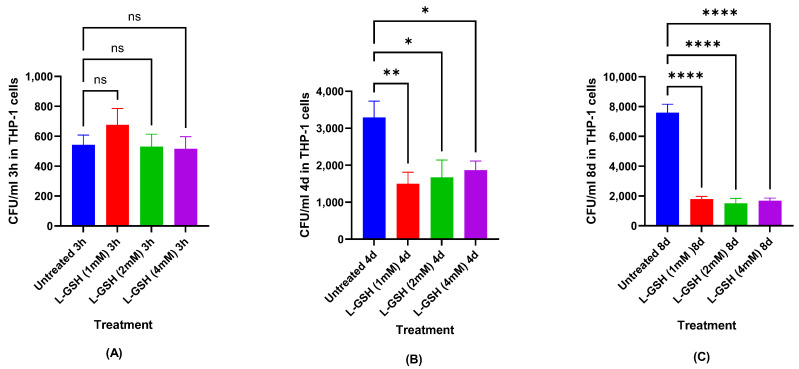
L-GSH reduces intracellular *M. avium* burden in THP-1 macrophages—low concentration range (1–4 mM). Differentiated THP-1 macrophages were infected with *M. avium* and treated with L-GSH at 1 mM, 2 mM, or 4 mM. Intracellular bacterial survival was quantified by CFU analysis. (**A**) 3 h post-infection; (**B**) 4 days post-infection; (**C**) 8 days post-infection. Data are presented as mean ± SEM from three independent experiments, each performed in triplicate (n = 3). Statistical analysis: one-way ANOVA. ns = not significant; * *p* ≤ 0.05, ** *p* ≤ 0.01, **** *p* ≤ 0.0001 compared to untreated control.

At 3 h post-treatment (Figure 4A), no significant differences in intracellular CFU were observed between L-GSH-treated macrophages and untreated controls across all concentrations. This indicates that L-GSH does not exert immediate bactericidal activity within the early post-infection window.

At 4 days post-treatment (Figure 4B), L-GSH produced significant reductions in intracellular bacterial burden. Treatment with 4 mM L-GSH resulted in the most pronounced reduction, with approximately three-fold lower CFU compared to untreated controls (*p* ≤ 0.0001). The 2 mM and 1 mM treatments also showed significant reductions, though to a lesser extent.

At 8 days post-treatment (Figure 4C), sustained and robust antimycobacterial activity was observed. All L-GSH concentrations significantly reduced intracellular *M. avium* CFU compared to untreated controls (*p* ≤ 0.0001 for all).

#### 3.4.2. High Concentration Range (10–40 mM)

**Figure 5:**
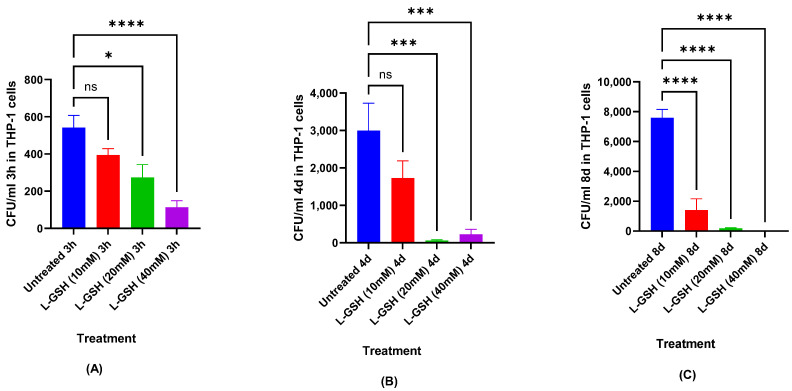
L-GSH reduces intracellular *M. avium* burden in THP-1 macrophages—high concentration range (10–40 mM). Differentiated THP-1 macrophages were infected with *M. avium* and treated with L-GSH at 10 mM, 20 mM, or 40 mM. Intracellular bacterial survival was quantified by CFU analysis. (**A**) 3 h post-infection; (**B**) 4 days post-infection; (**C**) 8 days post-infection. Data are presented as mean ± SEM from three independent experiments, each performed in triplicate (n = 3). Statistical analysis: one-way ANOVA. ns = not significant; * *p* ≤ 0.05; *** *p* ≤ 0.001; **** *p* ≤ 0.0001 compared to untreated control.

At 3 h post-treatment (Figure 5A), a significant reduction in intracellular *M. avium* CFU was observed at higher L-GSH concentrations (20 mM and 40 mM) compared to untreated controls (*p* ≤ 0.05 for 20 mM; *p* ≤ 0.01 for 40 mM). The 10 mM treatment did not show a significant reduction at this early point.

At 4 days post-treatment (Figure 5B), L-GSH produced marked reduction in intracellular bacterial burden. Treatment with 20 mM and 40 mM L-GSH resulted in greater reduction in *M. avium* viability when compared to 10 mM treatment.

At 8 days post-treatment (Figure 5C), sustained antimycobacterial activity was observed across all concentrations. The 40 mM treatment maintained near-complete bacterial clearance, while 20 mM and 10 mM also showed significant reductions compared to untreated controls (*p* ≤ 0.0001 for both).

These findings demonstrate that L-GSH effectively reduces intracellular *M. avium* survival in a concentration-dependent and time-dependent manner, with effects becoming more pronounced after sustained exposure (4–8 days).

## 4. Discussion

This study demonstrates that L-GSH possesses potent direct antimycobacterial activity against *M. avium* in axenic culture and effectively reduces intracellular bacterial survival in infected human THP-1 macrophages. The antimycobacterial effect was concentration-dependent and time-dependent, becoming more pronounced with prolonged exposure (4–8 days). Mechanistically, L-GSH induced membrane depolarization in *M. avium*, suggesting disruption of bacterial membrane integrity as a contributing mechanism. The MIC was determined to be 800 µM (0.8 mM).

### 4.1. Relevance to HIV-Associated M. avium Infection

The findings of this study have particular relevance to HIV-infected individuals, who are at increased risk for MAC infection due to both immune dysfunction and GSH deficiency. Multiple independent studies from our laboratory as well as other laboratories have demonstrated that immune cells from HIV-infected individuals exhibit a profound depletion of intracellular GSH compared to cells from healthy controls [27,28,29,30,31,32,33,34,35,36]. This depletion is consistently observed across macrophages, T lymphocytes, and erythrocytes, indicating a systemic redox imbalance rather than a cell-type-restricted phenotype [30,31,32,38,39,40,46,47,48].

Mechanistically, GSH deficiency in HIV infection has been linked to reduced expression of GCLC, GSS, and GSR, the key enzymes required for de novo GSH synthesis and recycling [30,31,32,33,34,35,36,37]. Morris et al. (2012) demonstrated that overproduction of pro-inflammatory cytokines in HIV-positive individuals leads to increased free radical production, which, combined with decreased expression of GSH synthesis enzymes, results in depletion of free GSH and contributes to loss of immune function [30,31,32]. Saing et al. (2016) further showed that GCLC, GSS, and GSR are significantly decreased in brain tissue from HIV-infected individuals, explaining increased susceptibility to severe forms of mycobacterial infection [37].

The functional consequences of GSH deficiency are profound and directly relevant to MAC susceptibility. Guerra et al. (2011) reported that GSH deficiency in T lymphocytes from HIV-infected individuals correlates with decreased levels of Th1 cytokines (IL-2, IL-12, IFN-γ) and enhanced growth of mycobacteria inside human macrophages [38,39,40]. Valdivia et al. (2017) demonstrated that HIV-positive individuals with low CD4 counts exhibit a Th2-biased immune profile (decreased IL-12, IL-2, IFN-γ; increased IL-6, IL-10, TGF-β), which compromises protection against intracellular pathogens like *M. avium* [41,42,43]. Ly et al. (2015) established a direct correlation between low GSH levels and increased susceptibility to mycobacterial infection through Th2-directed immune responses [44,45].

Importantly, restoration of GSH through supplementation has been shown to reverse these defects. Ly et al. (2015) demonstrated that 13 weeks of L-GSH supplementation in HIV-infected individuals significantly increased Th1 cytokines (IL-1β, IL-12, IFN-γ, TNF-α) and decreased free radicals and immunosuppressive cytokines (IL-10, TGF-β) [44,45]. Valdivia et al. (2017) confirmed these findings in a double-blinded study, showing that L-GSH supplementation for 3 months restored redox homeostasis and cytokine balance in HIV-infected individuals with low CD4 counts [41,42,43]. Morris et al. (2013) further showed that L-GSH treatment replenished rGSH levels in macrophages from HIV-infected individuals and correlated with decreased intracellular growth of mycobacteria [46,47,48].

The present study extends these observations by demonstrating for the first time, that L-GSH has direct antimycobacterial activity against *M. avium* independent of host immune cells. This dual mechanism, direct bactericidal activity plus immunomodulatory effects, positions L-GSH as a particularly attractive candidate for managing MAC infection in the context of HIV, where both pathogen-directed and host-directed strategies are needed. Nikjeh et al. (2025) specifically highlight GSH augmentation as a promising host-directed strategy for HIV-associated mycobacterial diseases, including MAC, noting its favorable ART compatibility [10,11,12].

### 4.2. Comparison with Prior Literature

The finding that L-GSH directly reduces *M. avium* viability extends previous observations from our laboratory and others regarding the antimycobacterial properties of GSH against *M. tb* [46,47,48,53,57]. While earlier studies focused primarily on immunomodulatory effects, the present study establishes that L-GSH has intrinsic bactericidal activity independent of host immune cells. This dual mechanism positions L-GSH as an attractive candidate for further development, particularly for immunocompromised populations where both pathogen control and immune restoration are critical.

The time-dependent nature of the antimycobacterial effect is notable. Minimal activity was observed at 3 h, whereas marked suppression occurred at 4 and 8 days. This delayed activity is consistent with the slow-growing nature of *M. avium* (doubling time approximately 24 h) and suggests that L-GSH may require sustained exposure to achieve maximal killing. Similar delayed antimycobacterial effects have been observed with other agents targeting mycobacterial cell envelope integrity [58,59].

The membrane depolarization data provides mechanistic insight into the direct antimycobacterial activity of L-GSH, but they do not establish causality. The DiSC_3_(5) assay measures loss of transmembrane potential through dye release and increased fluorescence; it confirms that membrane potential is disrupted in L-GSH-treated *M. avium* but does not identify whether depolarization is a primary event or secondary to other upstream effects. Several mechanisms may contribute. Liposomal GSH may interact directly with the mycobacterial outer membrane through its amphipathic lipid bilayer, disrupting glycopeptidolipid organization and increasing membrane permeability before GSH release occurs [60]. The significantly greater membrane depolarization produced by L-GSH compared with equimolar rGSH at 40 mM is consistent with a formulation-dependent effect, suggesting the liposomal carrier itself contributes to membrane disruption. The present data are best interpreted as establishing membrane depolarization as a candidate mechanism warranting further mechanistic investigation.

The apparent discrepancy between the broth MIC (800 µM) and the concentrations used in THP-1 experiments (1 to 40 mM) reflects intracellular pharmacokinetic barriers rather than a lack of dose–response logic. Effective intracellular delivery of liposomal contents to phagosomal bacteria requires membrane fusion and pH-dependent release steps that reduce the effective intracellular concentration to a fraction of the nominal extracellular dose. Future pharmacokinetic studies measuring intracellular GSH concentrations in L-GSH-treated macrophages will allow direct comparison of intracellular exposures to the broth MIC.

The ability of L-GSH to reduce intracellular *M. avium* survival in THP-1 macrophages is clinically relevant, as *M. avium* persists within macrophages where it is protected from many antibiotics [61,62]. L-GSH reduced intracellular bacterial burden in a concentration-dependent manner, with significant effects at 4 and 8 days but not at 3 h. The near-complete clearance observed at 40 mM after 8 days is encouraging, though this concentration may not be clinically achievable. The low concentration range (1–4 mM) also produced significant activity, with 4 mM representing approximately 5× MIC.

### 4.3. Clinical Translation Potential for HIV Co-Infection

Several features of L-GSH make it particularly attractive for HIV-associated MAC infection. First, L-GSH has a favorable safety profile, with no significant drug–drug interactions with antiretroviral agents reported in clinical trials [44,45,49,50,51]. Dawi et al. (2026) reviewed the safety of GSH supplementation across diverse populations, including individuals receiving complex antiretroviral and antitubercular regimens, and found that gastrointestinal discomfort was the most commonly reported adverse effect, with serious toxicities remaining rare [49]. This is a critical advantage over rifampicin-containing MAC regimens, which interact with protease inhibitors and non-nucleoside reverse transcriptase inhibitors [22,23,24].

Second, L-GSH addresses the underlying GSH deficiency that contributes to immune dysfunction in HIV, potentially restoring host immunity in parallel with direct antimycobacterial activity. Clinical studies have shown that L-GSH supplementation increases Th1 cytokine production, reduces inflammatory markers, and improves CD4+ T cell counts in HIV-infected individuals [41,42,43,44,45].

Third, liposomal formulation enhances oral bioavailability and cellular delivery, making it suitable for outpatient management [30].

### 4.4. Limitations

Several limitations should be acknowledged. All experiments were conducted in vitro using a single *M. avium* isolate and a single cell line (THP-1). While THP-1 cells are well-established, they may not fully recapitulate primary human alveolar macrophages. Future studies should validate findings using primary human macrophages and additional clinical isolates. The L-GSH concentrations used are supraphysiological; the MIC of 800 µM is more clinically relevant. Although prior work from our laboratory using ReadiSorb L-GSH in macrophage infection models at concentrations up to 40 mM did not identify significant cytotoxicity, we did not formally assess L-GSH cytotoxicity against THP-1 macrophages in this study [55,56]. Therefore, a formal dose-dependent cytotoxicity assessment using an established viability assay such as Thiazolyl Blue Tetrazolium Bromide (MTT), Lactate dehydrogenase (LDH) release, or trypan blue exclusion was not performed in the current study. Future experiments should confirm macrophage viability at all concentrations tested and present the results alongside intracellular CFU data. Until cytotoxicity data are available, the intracellular CFU reductions at 20 to 40 mM should be interpreted with caution. Finally, membrane depolarization experiments were performed in buffer, not within host cells.

A vehicle control using empty liposomes at equivalent lipid concentrations was not included in the antimycobacterial or membrane depolarization experiments. Liposomal lipid components, particularly cationic or zwitterionic phospholipids, can themselves interact with mycobacterial membranes and contribute to membrane perturbation [60]. Without an empty liposome control, the relative contribution of GSH versus the liposomal carrier to the observed antimycobacterial activity and membrane depolarization cannot be separated. This is an important limitation that should be addressed in follow-on studies. The greater activity of L-GSH compared with equimolar rGSH in the membrane depolarization assay (Figure 3B) suggests a formulation-dependent component, which could reflect GSH delivery enhancement, direct lipid-membrane interaction, or both. Additionally, while we have discussed the relevance to HIV infection, this study did not directly use HIV-infected cells or include HIV-specific controls. Future studies using HIV-infected macrophages or cells from HIV-infected donors would strengthen the translational relevance of these findings.

Furthermore, while DiSC_3_(5) demonstrated membrane depolarization following L-GSH treatment of *M. avium,* additional mechanistic studies are needed to strengthen the translational and biological significance of this finding. One such study is an Ethidium Bromide (EtBr) uptake assay, which would provide correlative evidence of cell envelope disruption by determining whether L-GSH-induced membrane depolarization is associated with increased membrane permeability in *M. avium*.

### 4.5. Future Directions

Formal cytotoxicity assessment of L-GSH against THP-1 macrophages and primary human alveolar macrophages using MTT, LDH release, and trypan blue exclusion assays at all concentrations tested (1 to 40 mM) should be completed before advancing to in vivo studies. Furthermore, the formal Clinical and Laboratory Standards Institute (CLSI) based MIC testing using visual endpoint or resazurin reduction is a priority for future work. In vivo studies using animal models of HIV-MAC co-infection (e.g., humanized mice or SIV-infected non-human primates) are needed to determine whether L-GSH reduces bacterial burden in the lung and prevents disseminated disease. Combination studies with standard antimycobacterial agents (azithromycin, rifampicin, ethambutol) and with antiretroviral agents should evaluate potential synergy and absence of antagonism. Pharmacokinetic studies are needed to determine achievable L-GSH concentrations in lung tissue and alveolar macrophages. Mechanistic studies using electron microscopy and lipidomics should further characterize membrane disruption. Using Transmission Electron Microscopy (TEM) would help determine if L-GSH physically fuses with or disrupts *M. avium’s* outer envelope. Additionally, testing membrane permeability is a necessary step to assess if L-GSH has a direct toxic effect on *M. avium*. Performing a comparative ROS quantitation assay would help determine indirect versus direct antimicrobial mechanism. To distinguish between indirect and direct antibacterial mechanisms, it would be beneficial to perform the ROS quantitation assay among these three groups: *M. avium* alone with L-GSH, macrophages alone with L-GSH, and infected macrophages with L-GSH. Finally, cytokine responses of L-GSH-treated, *M. avium*-infected macrophages from HIV-infected individuals should be characterized to confirm immunomodulatory effects in the relevant patient population.

## 5. Conclusions

L-GSH exhibits potent direct antimycobacterial activity against *M. avium* through membrane depolarization and reduces intracellular bacterial survival in infected THP-1 macrophages. Given the established GSH deficiency in HIV-infected individuals, driven by decreased expression of GCLC, GSS, and GSR, and associated with Th2-biased immune dysfunction, and the clinical need for novel MAC therapies with favorable safety profiles and minimal drug–drug interactions, these findings support further investigation of L-GSH as a host-directed therapeutic agent for HIV-associated *M. avium* complex infections.

## Figures and Tables

**Table 1:** Dose-dependent inhibition of *M. avium* by LGSH.

L-GSH Concentration	Mean % Growth Inhibition ± SD
600 µM	35.27 ± 4.92
*800* µM *(MIC)*	*67.81 ± 2.45*
1 mM	69.33 ± 2.33
2 mM	80.24 ± 1.50
4 mM	108.76 ± 0.67

*M. avium* (1 × 10^5^ CFU/mL) was treated with increasing concentrations of L-GSH in 7H9 medium for 4 days. Percent growth inhibition was calculated relative to untreated controls. Values >100% reflect OD reduction below baseline. Data are presented as mean ± SD from three independent experiments, each performed in triplicate (n = 3). The MIC was defined as the lowest concentration achieving consistent inhibition (SD < 10% of the mean), determined as 800 µM.

## Data Availability

The original contributions presented in this study are included in the article. Further inquiries can be directed to the corresponding authors.

## Abbreviations

The following abbreviations are used in this manuscript:

ADC	Albumin-dextrose complex
ANOVA	Analysis of variance
ART	Antiretroviral therapy
CCCP	Carbonyl cyanide m-chlorophenyl hydrazone
CFU	Colony-forming unit
CLSI	Clinical and Laboratory Standards Institute
DiSC_3_(5)	3,3′-dipropylthiadicarbocyanine iodide
EtBr	Ethidium Bromide
GCLC	Glutamate-cysteine ligase catalytic subunit
GSH	Glutathione
GSS	Glutathione synthase
GSR	Glutathione reductase
HIV	Human immunodeficiency virus
IFN-γ	Interferon-gamma
IL	Interleukin
LDH	Lactate dehydrogenase
L-GSH	Liposomal glutathione
MAC	*Mycobacterium avium* complex
MIC	Minimum inhibitory concentration
MOI	Multiplicity of infection
MTT	Thiazolyl Blue Tetrazolium Bromide
NTM	Nontuberculous mycobacteria
OD_600_	Optical density at 600 nm
PBS	Phosphate-buffered saline
PLWH	People living with HIV
PMA	Phorbol 12-myristate 13-acetate
rGSH	Reduced free glutathione
RPMI	Roswell Park Memorial Institute medium
SD	Standard deviation
SEM	Standard error of the mean
TGF-β	Transforming growth factor-beta
Th1	T helper type 1
Th2	T helper type 2
